# Systematic monitoring of needs for care and global outcomes in patients with severe mental illness

**DOI:** 10.1186/1471-244X-10-36

**Published:** 2010-05-25

**Authors:** Marjan Drukker, Jim van Os, Maarten Bak, Joost à Campo, Philippe Delespaul

**Affiliations:** 1 Department of Psychiatry and Psychology, School for Mental Health and NeuroScience MHeNS, Maastricht University, The Netherlands; 2 Division of Psychological Medicine, Institute of Psychiatry, De Crespigny Park, Denmark Hill, London, UK; 3 Integrated Care Division, Mondriaan, South-Limburg, John F Kennedylaan 301 Heerlen, The Netherlands

## Abstract

**Background:**

It was hypothesised that the introduction of tools that allow clinicians to assess patients' needs and to negotiate treatment (Cumulative Needs for Care Monitor; CNCM), would be associated with global outcome improvements in patients diagnosed with severe mental illness.

**Methods:**

The CNCM was introduced in one region in South Limburg (the Netherlands) in 1998 (REGION-1998) and in the rest of South Limburg in 2004 (REGION-2004). By comparing these two regions, changes after the introduction of the CNCM could be assessed (between-region comparison). In addition, a pre-post within-patient comparison was conducted in both regions.

**Results:**

The within-patient comparison revealed that global outcomes of psychopathology and impairment improved in the first 3-5 years after the introduction of the CNCM. The between-region comparison revealed an improvement in global psychopathology but not in global impairment in REGION-2004 after 2004, while there was no such improvement in REGION-1998.

**Conclusion:**

Systematic clinical monitoring of individual severe mental illness patients, in combination with provision of feedback, is associated with global improvement in psychopathology. More research is needed to determine the degree to which this association reflects a causal effect.

## Background

It is assumed that a person-based rehabilitation strategy informed by systematic and cumulative assessments of need for care and severity of symptoms may contribute to improved outcomes in patients diagnosed with severe mental illness (SMI). However, there is little empirical data to suggest that such a systematic, needs-based approach benefits individual patients.

SMI is best characterised as a complex combination of psychiatric, somatic and social needs. Most SMI patients are diagnosed with schizophrenia or psychotic disorder. Many have needs in the domains of self-care, accommodation, daytime activities and social contact [1], and have trouble finding employment [2-4]. Psychiatric care providers, particularly in continental European countries, have difficulties in systematically focusing on patients' needs and often select patients for available services [5,6]. The efficiency of services may improve when treatment plans are needs based [7,8]. However, needs-based care can only be achieved through individual assessments that personalise rehabilitation strategies. This contrasts with current practice, in which treatment planning mostly remains an implicit intuitive process.

The Cumulative Needs for Care Monitor (CNCM) introduces diagnostic tools that allow clinicians to explicitly assess patients' needs and to negotiate treatment with the patient [9]. Clinicians are trained to attend to the assessment of needs, in order to customise interventions in negotiation with the patient. For this purpose, CNCM feedback reports supply individualised information on needs and outcomes over time and on the position of the individual in a reference group. Two previous studies evaluated the validity, feasibility and effectiveness of the use of the CNCM in treatment. The first showed that need for care assessed with the CNCM predicted psychiatric inpatient but not psychiatric outpatient care consumption in the year following the assessment [10]. The second study showed that unmet needs in domains affecting the ability to live independently were associated with met needs in these domains at the next assessment, suggesting that clinical decisions were guided by CNCM feedback on treatment outcomes. On the other hand, unmet needs in the domain of psychopathology did not predict a met need in the same domain at the next assessment. This could have been expected, as patients with treatment-resistant syndromes are selected in services that attend the most needy individuals [1]. Although these initial findings are encouraging, more research is needed. Wiersma and colleagues, for example, concluded that more research is needed in order to make an informed judgement about the effectiveness of mental health care in reducing the number of needs [11].

### Purpose

The present research examined the hypothesis that the systematic use of the CNCM, as a needs-based systematic assessment tool, would impact on global outcomes. The aim of the analysis was to compare the first CNCM assessment with subsequent assessments (within-patient comparisons) and to compare two regions (between-region comparisons). It was hypothesised that results would depend on the treatment history of the patient (new in care, 2-3 years in care or chronic; hereafter 'level of chronicity'), because chronic patients are more likely to present with needs that are persistent as a result of lack of treatment response.

The analyses described in this paper were carried out as part of the E-Tail study (Efficiency of Tailor-made Psychiatric Rehabilitation), which was designed to evaluate the efficiency and effectiveness of the CNCM.

## Methods

Real-life monitor data that had been longitudinally collected within clinical practice were used for the present analyses.

### The Cumulative Needs for Care Monitor

The CNCM is used in mental health care in both inpatient and outpatient settings in South Limburg (pop. 660,000), which is an ethnically homogeneous area. The monitor was introduced in 1998 in one region (the city of Maastricht, pop. 122,000; hereafter REGION-1998) and was expanded in 2004 to the rest of South Limburg (REGION-2004 I and II) [9].

Mental health professionals (psychiatrists, psychologists, nurses, social workers) were trained to administer CNCM forms, which provide clinical case information for individual feedback as well as cumulative data for the database.

CNCM forms include various validated clinical instruments, such as the Camberwell Assessment of Need (CAN) [12,13], the Brief Psychiatric Rating Scale (BPRS) [14] and the Global Assessment of Functioning (GAF) scale [15].

### Global outcomes

The GAF is the fifth axis of the DSM IV; scores range from 0 (poor) to 100 (very good) [15,16]. The GAF used in the CNCM is divided into its psychopathology component (GAF-p, sample range 1-95) and its impairment component (GAF-i, sample range 0-97) [17]. The GAF is recognised as a valid and comprehensive measure of psychiatric mental health when used in routine clinical practice [18]. Good to excellent agreement between patients and clinician has been reported [16]. Nevertheless, GAF scores for SMI in the CNCM database have drifted upwards because professional carers rate their patients in comparison to their caseload rather than according to the criteria given in the manual [9].

### Brief Psychiatric Rating Scale

The Brief Psychiatric Rating Scale (BPRS) is a comprehensive instrument designed to measure the severity of symptoms [9]. Based on previous BPRS research, a confirmatory factor analysis was performed, using the CNCM data [9]. This confirmed the existence of four underlying constructs (also called the BPRS symptom dimensions): negative symptoms, positive symptoms, manic excitement and depression/anxiety [9].

### Subjects

There is a lack of consensus on the definition of SMI [19,20]. The intention is to include in the CNCM all patients from 'integrated care' departments that are treating patients with severe and long-lasting psychiatric illnesses. Thus, criteria for SMI were implicitly followed. However, CNCM forms are sometimes also used in non-SMI patients. To exclude patients who were not diagnosed with SMI, criteria to select SMI from the total CNCM patient group were predefined. These criteria were based not only on diagnosis, given that diagnosis is not always made at the first assessment. Criteria for SMI were [9]: (i) having a diagnosis of schizophrenia or psychotic disorder (DSM IV 295, 297 or 298), or having affective disorder (bipolar disorder: 296.4-8 or 301.13) or depression with psychotic features (296.14, 296.24, 296.34), (ii) scoring 15 or higher on the positive symptoms scale of the BPRS, or (iii) having a combination of low functioning (GAF < 45) and needs in at least two of the four following domains: accommodation, welfare benefits, alcohol and drugs. These four domains of need were *a priori *selected from the CAN. Accommodation and welfare benefits refer to the primary necessities of life; needs in these domains therefore indicate major problems related to independent living and justify inclusion in the SMI group. Needs in the domain of alcohol and drugs indicate comorbidity in the area of substance use, which suggests a shared underlying liability and impact on disability [9,21]. Because services for alcohol and drug addiction did not take part in the data collection until 2007, patients with a primary diagnosis of substance abuse disorder but no other psychiatric disorder were not included in the analyses.

As the definition of SMI was to a certain degree arbitrary and an absolute cut-off would have been too strict, patients who scored less than 45 on one of the two GAF scales and had a need in one of the four above-mentioned CAN domains were included in the analyses as moderate mental illness (MMI) patients. Not included in the analyses were patients with mild psychiatric symptoms representing common mental disorder (i.e. all patients who did not meet the above criteria). Note that the above-mentioned criteria (which were based on the expert opinion of psychiatrists working with the CNCM) were used not to define SMI in a population, but to exclude the non-SMI group from the CNCM population, given that diagnosis is not available for all patients.

The CNCM monitors treatment in the course of routine care and is part of the routine outcome monitoring required by insurers and health authorities in the Netherlands. The board of directors and executives of the participating care providers approved its application. The data can be used both for evaluative purposes and managerial decisions, and for anonymised group comparisons to generate scientific publications. Ethical committees in Maastricht, Utrecht and Groningen have confirmed that by law this type of data collection is not in their remit as long as patients are aware of the purpose (including the use of their data for scientific publications). During the interviews, the patients are asked to consent to this purpose and their consent is recorded.

### Dataset

All patients receiving mental health care, both inpatients and outpatients, are ideally assessed by their professional carers every year and upon every major change in treatment or setting (e.g. hospitalisation, start of a new treatment, discharge). BPRS and GAF are scored by clinicians [9]. The CAN combines the ratings from both patient and interviewer using a priori decision rules. These rules imply that per domain, the highest score is entered on the form. For example, a need for care with respect to drug problems that is acknowledged by the clinician but not by the patient is scored as a need. Similarly, if a patient views himself as problematically lonely, this is acknowledged in the score even if the clinician thinks that the patient's social network is sufficiently large and that contacts with, for example, the family are satisfactory [9]. A feedback report based on these data is sent to the professional carer to be used to negotiate the treatment plan with the patient. All CNCM assessments are also registered in a cumulative database. In keeping with current legal requirements, patients are informed that anonymised routine clinical data are used for the purpose of regional health services research and are given the choice to opt out; in the very few cases where patients do opt out, their data are excluded.

CNCM forms that had been administered in the event of any major change in treatment or setting (e.g. hospitalisation, start of a new psychiatric treatment, discharge) were excluded from the analyses (see methodological issues). From the second half of 2006, a GAF re-anchoring exercise was undertaken, teaching professional carers to score lower on the GAF (see methodological issues). Therefore, all data collected after July 2006 were excluded.

### Years since baseline

Years in care since baseline was an independent variable in most of the analyses (see below). It was calculated by subtracting the baseline date (in days) from the assessment date (in days) and dividing the result by 365.25. The integer function was then applied to this variable, so that only full years were counted (90-365 days = 0; 366-730 = 1; etc.).

### Statistical analysis

All analyses were performed using Stata 11 [22]. As patients were interviewed several times, yielding more than one observation per person (i.e. several records in the dataset), the assumption of independence of the observations for standard linear regression analyses was not met. Multilevel linear regression analysis is suitable for the analysis of such structured data [23]. The regression coefficients obtained from multilevel linear regression analyses can be interpreted in the same way as the estimates obtained from standard unilevel analyses. The two GAF scales and the four BPRS dimensions were the dependent variables in the multilevel linear regression models.

A within-patient comparison was carried out in the combined regions of REGION-1998 and REGION-2004. Years since baseline was the main independent variable, while sex, age group (15-20, 21-30, 31-40, 41-50, 51-60, 61-91), level of chronicity (i.e. new patients, 2-3 years in care or chronic), type of care (outpatient, sheltered housing, inpatient, assertive community treatment), and one variable that included information on both region and period (REGION-1998 before 2004, REGION-1998 after 2004, REGION-2004 I and REGION-2004 II) were added as covariates. All categorical variables were recoded into dummies (reference categories: age 21-30, new patients, outpatients, REGION-1998 after 2004). One *a priori *interaction term was also included in the model: years since baseline*level of chronicity. Quadratic terms were added to the model in order to test whether the association between years since baseline and outcomes deviated from linearity.

For the between-region comparisons, the main independent variables were region (REGION-1998 or REGION-2004), years since baseline and the interaction term REGION*years since baseline. If outcomes improved in the region where the CNCM had been introduced only in 2004 while outcomes remained stable in the same time period in the region where the CNCM had been in place since 1998, the hypothesis of effectiveness of the CNCM would be supported. In the between-region comparison, baseline was the first measurement after July 2004 (hereafter 'baseline 2004'). For patients who were not assessed in the second half of 2004, baseline 2004 was the first assessment in 2005 or even 2006. The interaction term (REGION*years since baseline) was added in order to model a difference in changes over time between REGION-1998 and REGION-2004. Sex, age group, level of chronicity and type of care were added as confounders. Categorical variables were again entered as dummies. The 3-way interaction term REGION*level of chronicity*years since baseline 2004 was *a priori *included (as were all accompanying 2-way interaction terms). If there was evidence for interaction, regression coefficients in all strata of the interacting variable were calculated using the Stata Lincom procedure.

When testing the 3-way interaction, alpha was set at 0.1. In all other comparisons (main effects and 2-way interactions), alpha was 0.05.

## Results

### Within-patient comparisons

A total of 739 patients had undergone between 2 (55%) and 9 (0.1%) assessments. The assessments were on average 23 months apart; 28% were 1 year or less apart, while 11% were more than 4 years apart. The mean age of patients at first assessment was 42 years (range 18-85 years). Of all patients, 27% were MMI rather than SMI, and 42% were female.

Baseline GAF impairment score was 50.5 and in the following 3 years the scores were 56, 54 and 57, respectively (table 1). Baseline GAF psychopathology score was 54 and in the following 3 years the scores were 59, 58 and 59. Pearson correlations between BPRS dimensions and GAF were between -0.37 and -0.58 (p < 0.001), except for depression/anxiety where correlations were around -0.30 (p < 0.001).

**Table 1:** Mean change in GAF scores per year since first CNCM measurement

				years since baseline as a categorical variable		years since baseline linear	
	years since 1^st ^assessment	n	Mean GAF (sd)	β	95% CI	β	95% CI
GAF impairment	0 (ref)	718	50.5 (14.8)	0			
	1	153	55.4 (14.6)	3.73**	1.66; 5.80		
	2	73	54.3 (16.5)	1.30	-1.54; 4.16	0.69**	0.17-1.22
	3	67	57.0 (15.4)	4.85**	1.87; 7.85		
	4-5 year	93	54.5 (16.5)	3.04*	0.11; 5.97		
	6-9 year	74	50.2 (16.3)	1.92	-1.80; 5.64		
							
GAF psychopathology	0 (ref)	722	53.5 (16.0)	0			
	1	157	58.8 (15.4)	3.67**	1.45; 5.88		
	2	73	58.3 (18.0)	1.54	-1.54; 4.62	0.24	-0.32;0.81
	3	68	58.7 (16.1)	3.59	0.36; 6.81		
	4-5 year	96	54.8 (17.6)	0.57	-2.55; 3.69		
	6-9 year	79	50.4 (18.0)	0.92	-4.63; 3.29		

95% CI = 95% confidence interval; only baseline and yearly evaluation assessments were included.

* p < 0.05 ** p < 0.01

Analyses showed that the association between years since baseline and GAF or BPRS was not linear. Therefore, years since baseline was included as a categorical variable, formatted as dummies (0 years = reference category, 1, 2, 3, 4-5, 6-9 years). There was no interaction between years since baseline and level of chronicity.

In the first 3 years after baseline, both global outcome GAF scales (impairment and psychopathology) showed an improvement, both before (data not shown) and after (table 1) controlling for confounders. There was also some evidence of improvement in years 4-5 after controlling for confounders, but only for the impairment scale (p = 0.057).

**Figure 1:**
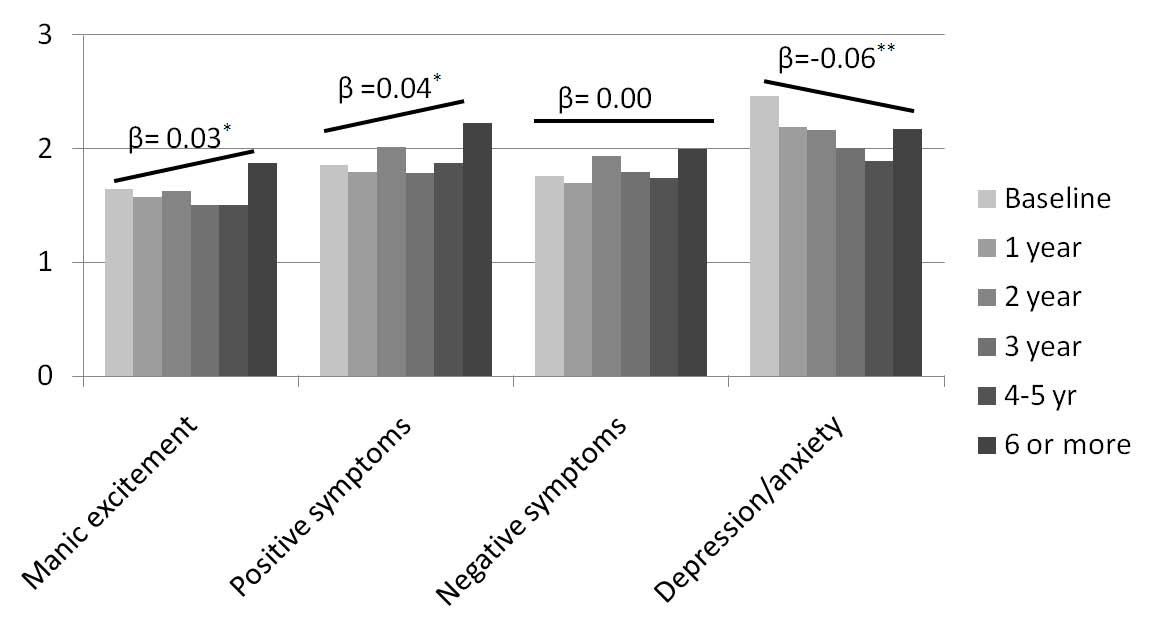
**Graphical presentation of the BPRS within subject results.** Bars indicate mean BPRS scores per stratum (crude) β = change controlled for age, gender, type of care, level of chronicity * p < 0.05 ** p < 0.001.

BPRS manic excitement and positive symptoms increased over the years (β = 0.03, p = 0.009 and β = 0.04, p = 0.003 respectively), while depression/anxiety decreased (β = -0.06, 95%, CI = -0.09; -0.04, p < 0.001, figure 1).

### Between-region comparisons

The CNCM database included 128 patients with multiple assessments between 2004 and July 2006. The assessments were, on average, 12 months apart (standard deviation between: 4.2); 83% were 1 year after baseline and 11% were 2 years after baseline. Mean age of the patients at baseline in 2004 was 42 years (range 18-84 years). Of all patients, 30% were MMI rather than SMI patients, and 40% were female.

In REGION-2004, baseline GAF scores were 49 and 48 (GAF-i and GAF-p, respectively) and scores increased in the next year to scores similar to those in REGION-1998. The numbers in the second year of follow-up were low (n = 6 in both REGION-1998 and REGION-2004).

**Figure 2:**
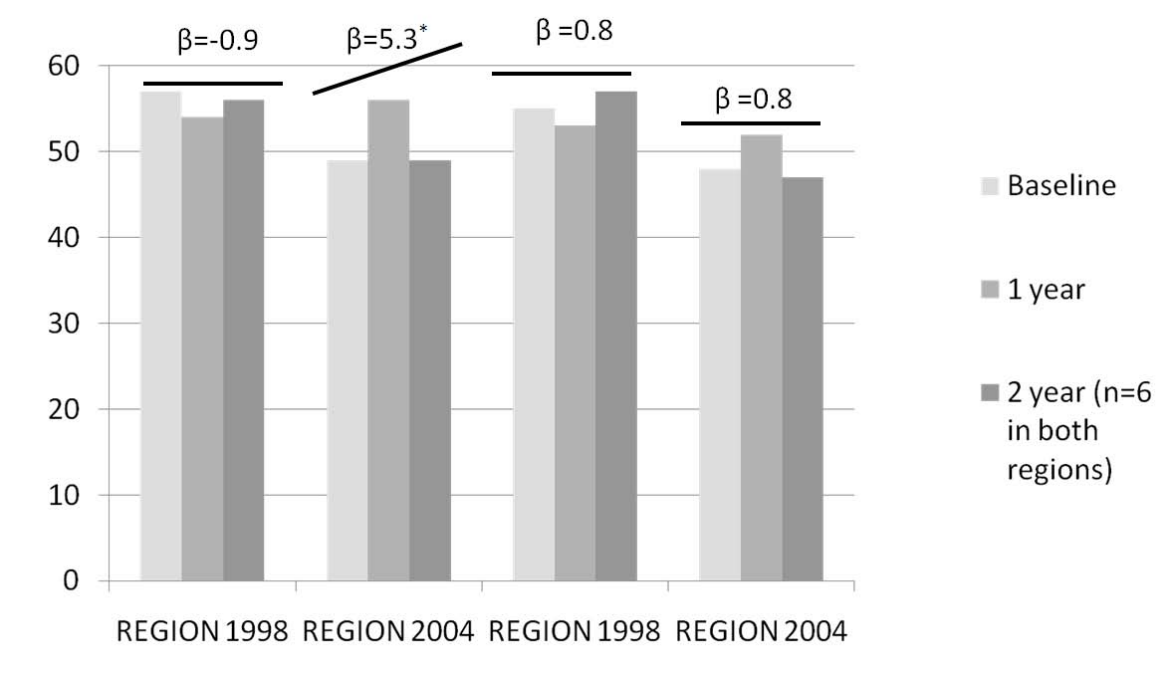
**Graphical presentation of the GAF between-region results.** Bars indicate mean GAF scores per stratum (crude) β = change controlled for age, gender, type of care, level of chronicity * p < 0.05

When the GAF scales were the dependent variable, none of the interaction terms that included level of chronicity was statistically significant. When years since baseline in 2004 was included as a linear variable (no evidence for a non-linear association), analyses showed an improvement in the GAF psychopathology scale in REGION-2004 after 2004, while there was no such improvement in REGION-1998 over the same time period (regression coefficient of interaction term = 6.47, p = 0.007; figure 2). The association between years since the baseline in 2004 and GAF impairment did not differ between the regions (regression coefficient of interaction term = 2.96, p = 0.22). Inpatients, patients in sheltered housing and patients in assertive community treatment scored lower than outpatients (data not shown).

Because the associations between years since baseline 2004 and BPRS dimensions were non-linear, years since baseline 2004 was included as a categorical variable (0, 1, 2-3 years). The 3-way interaction term REGION*level of chronicity*years since baseline 2004 was statistically significant when analysing manic excitement (χ^2 ^= 9.0, df = 4, p = 0.06, table 2) and depression/anxiety (χ^2 ^= 8.55, df = 4, p = 0.07).

**Table 2:** BPRS-sumscores in two regions in South Limburg, where baseline is the moment of the start of the CNCM in region 2004: descriptives and regression results (Only baseline and yearly evaluation assessments were included, to avoid bias)

	Descriptives				Regression results		
	**REGION**	**Baseline**	**1 year ** **follow-up**	**≥ 2 year ** **follow up**	**1 year vs ** **baseline^a^, ** **β (95% CI)**	**2-3year vs ** **baseline^a^, ** **β (95% CI)**	**Difference in difference = interaction term^b^**

number of subjects	1998	172 (175)	107 (107)	60(56)			
(assessments)	2004	393(380)	251(248)	205(196)			
							
BPRS manic excitement, mean (sd)	1998	1.32 (0.37)	1.62 (0.67)	1.58 (0.82)	0.38 (-0.15; 0.91)	0.49 (-0.45; 1.42)	1 yr β = -0.52 p = 0.11
*New patients*	2004	1.55 (0.88)	1.41 (0.71)	1.43 (0.52)	-0.13 (-0.49; 0.22)	0.04 (-1.00;1.07)	2-3 yr β = -0.34 p = 0.5
							
BPRS manic excitement, mean (sd)	1998	1.28 (0.49)	1.26 (0.42)	2.00 (1.78)	0.14 (-0.31; 0.58)	**0.62 ** (0.004;1.24)*	1 yr β = -0.17 p = 0.5
*2-3 years in care*	2004	1.53 (0.72)	1.49 (0.58)	1.24 (0.32)	-0.04 (-0.32;0.24)	-0.13 (-0.46-0.20)	**2-3 yr β = -0.75 ** **p = 0.04**
							
BPRS manic excitement, mean (sd)	1998	1.67 (0.79)	1.77 (0.87)	1.69 (0.83)	0.08 (-0.07; 0.23)	-0.03 (-0.22; 0.15)	1 yr β = -0.03 p = 0.7
*Chronic patients*	2004	1.64 (0.75)	1.63 (0.77)	1.79 (0.85)	0.04 (-0.06; 0.14)	**0.18 ** (0.07; 0.29)*	2-3 yr β = 0.21 p = 0.054
							
BPRS positive symptoms, mean (sd)	1998	1.99 (1.10)	1.91 (0.97)	2.12 (1.33)	-0.03 (-0.13;0.06)^b^	**0.12 ** **(0.02; 0.23)^c^**	χ^2 ^= 0.16 p = 0.92
	2004	1.88 (1.00)	1.78 (0.94)	2.00 (1.09)	-0.03 (-0.13;0.06)^b^	**0.12 ** **(0.02; 0.23)^c^**	
							
BPRS negative symptoms, mean (sd)	1998	1.84 (0.95)	1.80 (0.83)	1.90 (0.98)	0.02 (-0.14;0.18)	-0.09 (-0.29; 0.12)	**χ^2 ^= 8.95 p = 0.011**
	2004	1.72 (0.75)	1.71 (0.83)	1.94 (1.08)	0.02 (-0.08;0.13)	**0.36 ** **(0.11; 0.62)**	
							
BPRS depression/anxiety, mean (sd)	1998	2.40 (1.11)	2.96(0.89)	1.25 (0.35)	0.56 (-0.19; 1.33)	-0.30 (-1.65; 1.06)	**1 yr β = -1.23 ** **p = 0.008**
*New patients*	2004	2.69 (1.20)	1.80 (1.00)	2.44 (1.30)	**-0.67 ** (-1.17;-0.17)**	0.71 (-0.78; 2.20)	2-3 yr β = 0.14 p = 0.85
							
BPRS depression/anxiety, mean (sd)	1998	2.70 (1.11)	2.30 (1.15)	2.70 (1.27)	-0.22 (-0.84; 0.40)	0.62 (-0.27; 1.50)	1 yr β = -0.02 p = 0.96
*2-3 years in care*	2004	2.53 (1.16)	2.39 (1.12)	2.34 (1.27)	-0.24 (-0.64;0.17)	-0.17 (-0.64; 0.30)	2-3 yr β = -0.78 p = 0.13
							
BPRS depression/anxiety, mean (sd)	1998	2.30 (1.08)	2.31 (0.98)	2.45 (1.16)	-0.004 (-0.22;0.21)	0.05 (-0.22; 0.31)	1 yr β = 0.14 p = 0.27
*Chronic patients*	2004	2.51 (1.18)	2.34 (1.06)	2.44 (1.09)	**-0.15 ** (-0.29;-0.006)*	-0.00 (-0.15; 0.15)	2-3 yr β = -0.05 p = 0.75

^a^After controlling for confounders

^b^Is the regression coefficient in REGION-2004 significantly different from the regression coefficient in REGION-1998?

^c^Because the interaction region * (year since start in REGION-2004) was not statistically significant, this term was deleted from the regression model and, therefore, differences between baseline and follow up are exactly the same in both regions.

*p < 0.05

**p < 0.01

Both in new and in chronic patients, there was a decrease in depression/anxiety in REGION-2004, while there was no such decrease in REGION-1998 (table 2). However, this was only in the first year after baseline. Patients in care for two to three years in REGION-2004 scored lower on manic excitement than those in REGION-1998, but this was because of an increase in manic excitement in REGION-1998.

In the models without 3-way interaction (positive symptoms and negative symptoms), only the negative symptoms dimension showed interaction between region and years since 2004 (table 2). In REGION-2004, negative symptoms increased 2-3 years after baseline (β = 0.36 p = 0.005), while in REGION-1998 negative symptoms remained rather stable (table 2).

## Discussion

The results showed an improvement in both the psychopathology and the impairment global outcomes when follow-up measurements in the first three to five years were compared with the first assessment ever (within-person). The between-region comparison showed that this improvement was observed only in the region where the CNCM had been introduced in 2004, although this did not apply to the global impairment outcome. Thus, the results suggest that while patients may improve in psychopathology, social impairments may lag behind. This improvement in psychopathology was mainly visible in the depression/anxiety domain of the BPRS.

The within-person comparison included more observations and covered a longer time period, and was therefore likely to have more statistical power than the between-region comparison. Power may have been too low to show a consistent effect across comparisons, also because CNCM data are collected as part of routine clinical practice and are therefore likely to be 'noisier' than research data. In addition, the improvement in the psychopathology domain may be a precursor of a subsequent reduction in impairment. The improvement in symptoms may not always have been sufficiently large to result in a consistent change in impairment. Thus, gains on the GAF impairment scale may be found after more years have elapsed since implementation of the CNCM in REGION-2004. This assumption is supported by the present within-person results, which show an increase in the GAF impairments scale but no longer in the GAF psychopathology scale 4-5 years after baseline.

However, in the within-patient comparison, patients seem to be worse off in the later years. A likely reason is the selection of the most severe patients in these years, due to a selective discharge of less severe patients and their transfer to non-psychiatric carers who do not use the CNCM (GPs, social workers or residential care workers). In a sensitivity analysis of the BPRS within-patients analyses, in which only the first five years after baseline were included, there was no longer an association between years since baseline and manic excitement and positive symptoms, while the reduction in depression/anxiety was stronger (β = -0.09 95% CI = -0.12; -0.04 p < 0.001). In the GAF analyses, the linear gradient was stronger after including years 1-5 only (β = 1.03 p = 0.001 95% CI = 0.41-1.64 and β = 0.63 p = 0.06 95% CI = -0.03-1.28, respectively).

One of the observed mechanisms for better functioning may be improved patient-carer communication through systematic use of the CNCM and discussions on patient needs. A French study showed that communication between patient and professional carer can be beneficial [7,24]. The use of the 2-COM (a simple patient-reported questionnaire on 20 perceived need domains) in treatment plans was associated with higher satisfaction with care at the 12-month follow-up, and treatment change was more likely in patients with more reported needs. A multicenter study also showed associations between clinician use of DIALOG (a tool to discuss 11 domains of needs) and improvement in quality of life and unmet needs for care after 12 months, but no change in symptom levels [8]. The inconclusive result with respect to symptom levels is in agreement with the between-region findings with respect to GAF psychopathology. The authors stressed the positive implication of this finding, namely that quality of life can be improved even when symptoms remain unchanged [8]. Neither of the above-mentioned studies included functioning or other global outcomes [8,24].

It was unfortunately not possible to study the pathways by which the CNCM is associated with improved functioning. It is possible that what mediates this effect is the professional carer's attention to the patient and to the whole range of the patient's problems, rather than a change in treatment itself. This has been called the Hawthorne effect [25]. In RCTs, researchers try to eliminate this factor, because patients do not get the same amount of attention in real-life clinical practice. The CNCM, however, is not a research setting but part of real life, and therefore the Hawthorne effect represents a desirable outcome rather than a nuisance factor. In addition, the changes in patients' functioning can be a consequence of differences in the organisation of care after the introduction of the CNCM. The CNCM was developed to change health care, and it is beyond the scope of the analyses to disentangle aspects that co-occurred at the moment of introduction.

### Methodological issues

The strength of the present study is the unique data collection, as real-life observational data were obtained longitudinally within the treatment process. The longitudinal design resulted in assessment of need for care and functional outcomes at various moments over time.

However, the present study also has its limitations. First, only patients who had been assessed at least twice were included in the analyses. Patients who had been assessed only once, either because they were new to the service or had been discharged before the second assessment, showed less severe symptoms. Thus, results are representative only of the more severe subgroup within the SMI patient group.

Second, because clinicians have limited time to fill in CNCM forms, brief instruments were selected. A full research paradigm might have opted for more extensive instruments. The GAF, which in our application consists of only two ratings (psychopathology and impairment), is recognised as a valid and comprehensive measure of social functioning when used in routine clinical practice [17,18] and in research [17,26]. However, it has been reported that reliability is only fair when the GAF is scored by clinicians rather than researchers [27]. In the CNCM region, professional carers persistently scored higher than the manual prescribed [9]. The GAF is intended to be a self-explanatory instrument and raters are almost never trained in its use. The actual knowledge of the instructions is poor and raters are often misled by confusingly defined anchors. In addition, GAF instructions may be incomplete [16]. Nevertheless, research indicates that precision of GAF-scores at the group level is sufficient [27]. The relatively large number of patients in the CNCM database makes analysis of changes over time in a group of patients more reliable than the individual crude scores, and therefore validity is not at risk in these group-level comparisons. A sensitivity analysis of the within-subjects analyses using data collected after all participating clinicians had received extensive training in GAF scoring (i.e. after July 2007) yielded very similar results. In the between-region analyses, numbers were lower and the second follow-up year (n = 6 in both regions) may have yielded unreliable results. In addition, overall measures such as the GAF have been reported to be more sensitive to change than symptom dimensions [28]. This is in agreement with the present results: associations with GAF scores were stronger compared to the four BPRS dimensions.

Finally, MMI patients were included in the analyses because they differ only marginally from SMI patients. Post-hoc analyses that excluded this MMI group yielded very similar results. In the within-patient analyses, the results on GAF impairment were stronger.

## Conclusions

The present results suggest that the use of the CNCM in treatment has the potential to improve global outcomes. However, the results need to be replicated.

## Abbreviations

BPRS: Brief Psychiatric Rating Scale; CAN: Camberwell Assessment of Need; CNCM: Cumulative Needs for Care Monitor; GAF: Global Assessment of Functioning; MMI: Moderately Mental Ill; sd: Standard Deviation; SMI: Severe Mental Illness.

## Competing interests

The authors declare that they have no competing interests.

## Authors' contributions

MD performed the analyses and wrote the paper. JvO, MB and PhD contributed during interpretation of the analyses and edited various drafts of the paper. JvO and PhD are scientific coordinators of the CNCM. JaC implements the CNCM in his region/institution and edited the final draft of the paper. The final version has been read and approved by all authors.

## Pre-publication history

The pre-publication history for this paper can be accessed here:

http://www.biomedcentral.com/1471-244X/10/36/prepub
